# Practical Notes and Formulæ

**Published:** 1877-12-20

**Authors:** 


### Practical Notes and Formulæ.

How to Take Castor Oil Without Tasting It.—Dr. Z. B. Herndon, of Richmond, Va., favors us with the following:

Having reference to the relative specific gravity of water, whisky and oil, this nauseous dose can be so clothed with the mixture of whisky and water as to be swallowed without coming in contact with the mouth and palate—

Into a small glass put from one to two tablespoonsfuls of spirits; add one drop of oil, which, being heavier than whisky, sinks to the bottom; now add water, drop by drop, until the oil rises half way to the surface of the mixture; at this point put in the dose of oil, and it will occupy a central position in the form of a sphere; take at one swallow, and no taste of oil can be detected. The advantage of this method can not be over-estimated, where the stomach is delicate, or the patient has a preconceived idea that the oil can not be kept down.

Chloral and Alcohol.—A German physician reports that he has, on several occasions, observed considerable excitement after a dose of chloral. Anxiety and turgescence of the face, contracted pupil and rapid pulse (100 to 130) lasted two or three hours. The next day headache and lassitude were complained of. He found, on careful inquiry, that in every case in which these symptoms appeared, the patient had taken some alcoholic bev–erage shortly before the dose of chloral.

It seems desirable that this point should be observed by others. It is not at all unlikely that some of the evil effects attributed to chloral may be due to such a perturbing agent as alcohol. Physicians should caution their patients against the use of alcoholic liquors while being treated with chloral.—*Jour. Chem.*

Carbolic Acid in Diarrhœa.—Dr. Palmer says (in *Med. Rep.*): "Recently I have employed carbolic acid in the treatment of diarrhœa, both acute and chronic form of the disease, with signal success. It is given in one or two drop doses, largely diluted with water, from two to four hours apart. It controls pain, and corrects the fetor of the discharges, and otherwise cures the disease. If severe pain be present, and the discharges profuse, opium and creta preparata, with astringents, may be alternated with it. But my experience is that carbolic acid, in most case, is all that is required."

Freckles.—*New Remedies* gives the following:

R. Zinci sulphocarbol, 2 parts.
Glycerine, 25 parts.
Aq. rosæ, 25 parts.
Spiritus vini rect., 5 parts.

Dissolve and mix. The freckled skin is to be annointed with this twice daily, the ointment being allowed to stay on from one half to one hour, and then washed off with cold water. Anæmic persons should also take a mild ferruginous tonic. In the sunlight a dark veil should be worn.

It is also said that powdered nitre, moistened with water, and applied night and morning, will soon remove all traces of freckles.

Lime in the Eye.—The evil effects of lime in the eye are well known. Plasterers and whitewashers not unfrequently having their eyes seriously injured, if not destroyed, by the caustic power of the lime. Wells says: "If the patient is seen soon after the accident, an effort should be made at once to neutralize and wash out the lime by a weak solution of vinegar, with a free use of the syringe. Afterwards, cooling and anodyne lotions, and general antiphlogistic treatment, should be adopted.

Burns.–We have suggested to Dr. Potter to use on burns and scalds, bromochloralum diluted one part to four of water, and applied as cold as itcan be made with ice. We use it entirely in our works; the aluminium acts upon the denuded surface, and the chlorine allays the inflammation. Cloths should be quite wet, and changed as soon as warm. We have never seen a case that did not yield promptly, and what is of great value no suppuration takes place, and no scar results.—*Jour. Mat. Med.*

(1)—ELIXIR OF WILD CHERRY BARK may be made as follows;

Fluid extract of wild cherry........4 ounces.
Simple elixir.......................12 "

Mix and filter. Several other formu las in which the preparation is obtained directly from the bark are to be found in the *Druggists' Circular* of January, May and July, 1874, pages 33, 89 and 123.

(2)—The following is probably what you want:

*Compound Syrup of Wild Cherry and Tar.*

Syrup of wild cherry........10 fluid ounces.
Syrup of tar..................4 " "
Syrup of tolu.... ......... ....2 " "

—*Drug. Circular.*

RICORD'S COUGH-PILLS.—

Morphiæ hydrochloratis....gr. v;

Extracti hyoscyami........gr. viij;

Rad. belladonnæ pulv...... }

Rad. glycyrrhizæ pulv..... } aa gr. xlv;

Mellis.... ............ }

Balsami tolutani...........gr. lxxv;

Ol. theobromæ............gr. lxxv;

Make into one hundred pills. Each contains one twentieth of a grain of hydrochlorate (muriate) of morphia.

Dose—One pill every five or six hours, in chronic bronchitis accompanied with cough.—*New Remedies.*

RUM STOMACH OR FLATULENT DYSPEPSIA.—For this form of dyspepsia, which is due to a torpid or a semi-paralyzed condition of the muscular coat of the bowels, and usually attended with constipation, the following is an excellent formula:

R. Tinc. Nucis Vomicæ.............gtt. v.

Com. Tinc. Gentian ... ............dr. j.

Tinc. Capsicum..................gtt. x.

To be taken before meals.

CANADA BALSAM AS AN EXCIPIENT FOR PILLS.—Dannecy proposes, as an excipient that will preserve pills for an indefinite period, a mixture of one part of wax and three of Canada balsam.

SHAVING SOAP.—We do not believe in shaving, but if it must be done, use the following soap, suggested by the *Druggists' Circular* as best for the skin:

R. White Wax, }

Spermaceti, } aa ½ oz.

Almond Oil, }

Melt, and before cooling rub in two cakes of Windsor Soap, which have previously been reduced to a paste with a small quantity of rose water. Lather upon the face with the brush.

ELIXIR CHLOROFORMI.—[*Dr. Hartshorne's Chloroform Paregoric.*]—

Chloroform...................12 fluid drachms.
Tincture of opium........12 " "
Tincture of camphor .....12 " "
Arom. spirit of ammonia..12 " "
Oil of cinnamon..........20 minims.
Brandy.................2 fluid ounces.

Mix. Dose, half a fluid drachm or less, in spasmodic affection of the stomach, cholera, etc.—*Drug Circular.*

CRYSOPHANIC ACID IN PSORIASIS.—An ointment of crysophanic acid in conjunction with the internal use of the syrup of the iodide of iron has been found an efficacious treatment for psoriasis and other obstinate skin affections in St. George's Hospital:

R. Crysophanic acid................dr. ss.
Lard.......................... ..oz. j.

M.

HAIR WASH.—A good simple "oily hair wash" can be made by the following formula:

Bay rum.........................4 ozs.
Glycerine.........................1 "
Olive oil..........................1 "

If not "oily" enough there is a very easy way out of that difficulty.—*Chemist & Druggist.*

RANULA.—Dr. Panas has frequently succeeded in curing ranula by the injection into the tumor of from four to ten drops of a concentrated solution of the chloride of zinc.

COPAIBA RESIN IN ASCITES. — Dr. Glynn has successfully used 20 grains, every four hours, in a case where other remedies failed. It was given in rectified spirits and a little mucilage.

ENLARGED TONSILS—

R. Glycerini ............................oz. ss.
Tannic accid.....................gr. xx.
Carbolic acid.......................gtt. x. M.

Mop the tonsils night and morning, and give internally—

R. Syr. Sarsaparilla......................dr. iij.
Iodid. Potass .....................oz. j. M.

and give from half to a teaspoonful, according to the age of the child.

Another:

R. Glycerini ............................oz. ss.

Tinc. iodine.........................dr. ij. M.

Mop the tonsils once daily, and give internally syrup of the iodide of iron gtt. x. to xx., three times daily.

NITRIC ACID, SENNA AND TARAXICUM, AS A LIVER REGULATOR.—To *liver disease*, *constipation* and *dyspepsia*, the following combination, from *Tanner's Index*, seems admirably adapted:

R. Dilute Nitric acid....................gtts. 90.
Spts. Nitre .............................dr. ij.
Fluid ext. taraxicum.....................oz. jss.
Tinc. Sennæ ...........................oz. iv.
Infusion of gentian com., sufficient to make.....................oz. viij M.

One-sixth part twice or thrice daily. [The dose seems too large in respect to the nitric acid.—ED.]

SYRUP OF HYPOPHOSPHATE OF SODIUM, may be prepared thus: (*Amer. Jour. Phar.*) Dissolve 5 grammes of the salt in 445 grammes of simple syrup, and add 50 grammes of orange flower syrup. A tablespoonful weighing 20 grammes contains 0.20 grammes (3 grains) of sodium hypophosphite.

CHLORIC ETHER is the name which was at one time applied to solutions of chloroform in alcohol. The officinal *spiritus chloroformi*, made according to the formula corrected as above, is intended to replace the varying solutions formerly employed under the name of chloric ether.—*Drug Circular.*

FOR BED SORES.—R. Caoutchouc, or gutta percha, a few chips or thin pieces, and dissolve in chloro orm. To be painted over superficial excoriations, or bed sores, as a protection against friction, etc.

FOR VERMIN.—

R. Mercurial ointment.................oz. i.

S. Use a small quantity for two or three applications.

Another:

R. Carbolic acid..........................dr. j.
Glycerini.................................oz. j.
Water.....................................oz. viij. M.

Use twice daily for three or four days.

CHRONIC HEPATIC FFECTIONS. The following preparation is well adapted to all chronic disorders of the liver, especially when complicated with rheumatic or neuralgic troub e:

R. mmonia Muriatis
Ext. Taraxaci .........................oz. ss
Aquæ.....................................oz. vj.

Dessert-spoonful three times a day.

SALICYLIC ACID IN PERTUSSIS.—In Petersburg *Med. Woch.* it is said that a 2 per cent. solution of salacylic acid inhaled for five minutes every evening in pertussis, effects an immediate influence, a cure resulting on an average in about two weeks.

TONIC, LAXATIVE AND ANODYNE.—The following, by Dr. Wm. Pepper, is recommended as an excellent tonic, laxative and anodyne:

R. Aloes, gr. 1.

Cannabis Ind., gr. ¼.

Pulv. Sulphur. Ferri, gr. 1.

F. 1 Pill to be taken at bed time.

SPIRITUS CHLOROFORMI.—The formula of spiritus chloroformi, (says the *Drug Circular*), should read *alcohol* instead of diluti alcohol as given in U. S. Dispensatory. Thus.—

R. Purified chloroform....1 troy ounce.
Stronger alcohol.........12 fluid ounces.

PUERPERAL FEVER.—For puerperal fever, bring the pulse under the decided influence of veratrum viride, and keep it there. Give opium enough to relieve pain, and use turpentine steepes to the abdomen.

CARBOLIC ACID SPRAY.—

R. Carbolic acid..........................gr. j.

Wa er.....................................oz. j. M.

To be used with a spray apparatus in affections of the nares, pharynx, etc.

RED LOTION OF HOSPITALS.—The common Red Lotion of hospitals, so useful for strumous and other ulcers, is composed as follows:

R. Zinci Sulphatis.................gr. xvj.
Spts. Rosmarini..............
Tinc. Lavender, com aa..........dr. ij.
Aqua...........................oz. viij.
M. —*Tanner.*

TANNIN GARGLE.—

R. Tannic acid .................... gr. xx.
Whisky ...........................oz. j.
Camph. water. ................oz. viij. M.

In relaxation of uvula, sore throat, etc.

GARGLE FOR FOUL ULCERS.—

R. Liquoris Sodæ Chloratæ..........dr. vj.

Aqua. .........................oz. viij. M.

To be frequently used.—*Ib.*

IODINE INHALATION.—

R. Tinct. Iodine..................gtt. xxx.
Boiling water.... .................. oz. iv. M.

The vapor to be cautiously inhaled in laryngeal phthisis, diptheria.—*Ib.*

TO CHECK SECRETION OF MILK.—

R. Ext. belladonna................ ..dr. j.
Glycerini........................oz. ss. M.

To be used upon the breast to check secretion of milk, with caution as to the child. Also useful as an application in neuralgic pains.

TO KEEP LEECHES.—A correspondent of the *Phar. Jour.* (London) says that it is a mistake to put fresh water to leeches. They do better in stale water. "By putting them in fresh water you starve them."

EPILEPTIC HYSTERIA.—

R. Bromide potass.............gr.xx.
Tinct. gelseminum.........gtt.xv.
Water. ...................oz.ss. M.

Give at a dose, and repeat in one hour if spasms continue.

TURPENTINE INHALATION.—

R. Oil of Turpentine................oz. j.
Boiling water.................oz. viij. M.

To be used with a common inhaler.

FOR TAPE WORM.—Drink freely of a strong tea of pomegranate bark, fasting.

FOR CHRONIC RHEUMATISM.—Fl. ex. asclepias incarnata.

